# Unraveling the Enigma: Exploring the Periphery’s Influence in Alzheimer’s Pathophysiology—Cause or Consequence?

**DOI:** 10.5152/eurasianjmed.2025.24592

**Published:** 2025-04-04

**Authors:** Gamze Sönmez, Yiğit Yazarkan, Özlem Erden Aki, Ebru Bodur

**Affiliations:** 1 Department of Medical Biochemistry, Hacettepe University Faculty of Medicine, Ankara, Türkiye; 2 Undergraduate, Hacettepe University Faculty of Medicine, Ankara, Türkiye; 3 Department of Psychiatry, Hacettepe University Faculty of Medicine, Ankara, Türkiye

**Keywords:** Alzheimer’s disease, gut–brain axis, liver–brain axis, pathophysiology, systemic disease

## Abstract

Alzheimer’s disease (AD) remains a formidable challenge, impacting individuals, families, caregivers, and society. Despite being identified over a century ago, effective drug treatments for AD remain elusive, with numerous clinical trials failing to produce meaningful results. The pathological hallmarks of AD, including the accumulation of beta-amyloid plaques and tau protein tangles, are well-established contributors to cognitive decline. However, recent research has raised questions about the efficacy of therapies targeting these abnormalities. Emerging evidence suggests that AD should not be viewed purely as a brain-centered disease but as a systemic condition involving complex interactions between the brain and peripheral organs. While the mechanisms linking peripheral processes and AD pathology remain unclear, studies indicate that these systems may contribute to or be affected by the disease. Recognizing AD as a heterogeneous disorder with systemic implications opens new opportunities for therapeutic innovation. Multimodal therapies targeting both central and peripheral aspects of AD pathology—such as amyloid-beta deposition, neuroinflammation, and systemic dysfunction—hold promise for slowing disease progression. This review aims to critically assess the current understanding of AD pathology, with a particular focus on the peripheral system’s involvement and its interplay with the brain. Additionally, it will explore novel therapeutic strategies and emphasize the importance of interdisciplinary collaboration to advance our knowledge and develop effective treatments.

**Main Points**

- Alzheimer’s disease (AD) is characterized by the accumulation of beta-amyloid plaques and tau protein tangles in the brain, leading to cognitive decline.
- There are ongoing questions about the effectiveness of targeting beta-amyloid plaques and tau protein abnormalities in AD treatment.
- Alzheimer’s disease is increasingly recognized as a systemic condition, involving communication between the brain and peripheral organs.
- Recognizing AD as a heterogeneous disorder with systemic implications suggests new opportunities for therapeutic strategies.

## Introduction

Alzheimer’s disease (AD), a complex neurodegenerative condition, presents one of the most daunting challenges of our time. Its impact is profound, not only on those directly affected but also on their families, caregivers, and society. Named after Dr. Alois Alzheimer, who first described it in 1906, it is characterized by progressive cognitive decline, memory loss, and behavioral changes.^1^ It is the most common cause of dementia, accounting for approximately 60%-70% of cases worldwide. The projected number of individuals affected by AD dementia in the United States in 2010 is expected to almost triple by the year 2050.^2^ The pathology of Alzheimer’s involves the buildup of beta-amyloid plaques and tau protein tangles in the brain, leading to neuronal damage and eventual cognitive impairment.

The pursuit of effective drug treatments for AD has been fraught with numerous disappointments and setbacks, leading to a disheartening series of drug failures in clinical trials, highlighting the complexity and elusive nature of AD pathology. Many promising drug candidates targeting amyloid-beta (Aβ) plaques or tau protein abnormalities, hallmark features of AD, have failed to demonstrate significant efficacy in late-stage clinical trials. The failure of these drugs has prompted researchers to ask: Do we truly understand the pathophysiology of AD, or are we just circling the same targets (Aβ plaques and tau protein abnormalities) and constantly arriving at the same point? Answering these 2 questions is important for developing alternative therapeutic strategies to address this growing global health crisis.

There is a growing consensus that AD is a systemic condition characterized by communication between the brain and peripheral organs. Studies on risk factors imply that peripheral processes might initiate or worsen the neurodegenerative process to some extent. Conversely, certain peripheral manifestations could be the consequence of AD processes, either through dysfunctional central nervous system (CNS) regulation of peripheral processes or behavioral changes leading to systemic effects. Although there are numerous ongoing studies, it remains unclear whether systemic processes trigger AD or if AD itself creates systemic effects. It would be accurate to describe AD as a heterogeneous metabolic and neurodegenerative disorder accompanied by systemic findings. In this context, exploring the role of peripheral organs in AD pathophysiology holds promise for uncovering novel therapeutic targets and strategies aimed at combating this devastating disease. This paper delves into the emerging evidence linking peripheral organ dysfunction to the pathogenesis of AD, shedding light on new avenues for research and therapeutic intervention. It also highlights the dysregulation of intracellular homeostasis and energy metabolism in the pathogenesis of AD, positioning them as both initiators and outcomes of the disease process.

## Pathophysiology of Alzheimer’s Disease

Neuritic plaques, formed by clumps of amyloid-beta proteins, and neurofibrillary tangles, resulting from accumulated tau proteins, are the characteristic biological indicators of AD.^3^ Early stage of AD is recognized as the cellular phase. Changes in neurons, microglia, and astrocytes contribute to the gradual progression of the disease before cognitive decline becomes apparent. Factors such as neuroinflammation, alterations in blood vessels, aging, and dysfunction of the glymphatic system occur upstream or alongside the accumulation of Aβ in this cellular disease context. Amyloid-β triggers the spread of tau pathology through an unknown mechanism, which is correlated with the presence of markers of necroptosis in neurons exhibiting granulovacuolar degeneration.^4^ There is a synergistic relationship between tau and Aβ; they work together to damage mitochondria and initiate inflammatory cascades in non-neuronal cells such as microglia. These 2 molecules play significant roles in the pathogenesis; however, treatments targeting these molecules have not yielded sufficient results. Also, activated glial cells represent another histopathological hallmark indicating neuroinflammation in regions affected by AD due to the accumulation of Aβ and hyperphosphorylated tau, which is a normal biological response of the CNS.^5^

**Figure 1.**
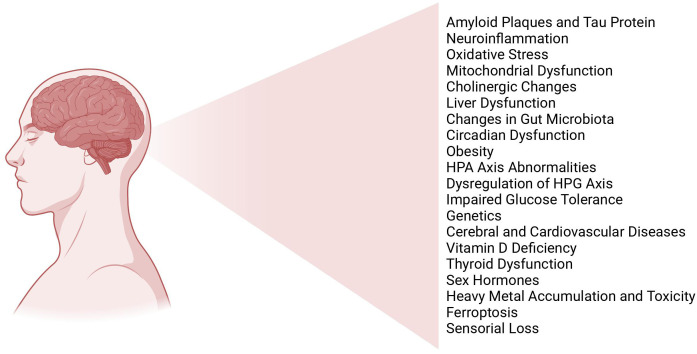
Different mechanisms that have a role in Alzheimer’s disease. HPA, hypothalamus–pituitary–adrenal; HPG, hypothalamic–pituitary–gonadal.

Different mechanisms have been proposed to explain the pathophysiology aside from Aβ and tau (Figure 1). We can categorize these mechanisms simply as genetic and non-genetic factors. Extensive genome-wide studies reveal that over 70 genes or genetic regions play a role in AD. Identifying genes is crucial for understanding molecular pathways in the pathogenesis of AD. So far, the molecular pathways identified through gene studies include lipid transport and metabolism, cytoskeletal function, and axonal transport, hippocampal synaptic function, immune system/inflammation, and cell migration.^6^ However, a significant hurdle to advancement is that the majority of genetic data stem from non-Hispanic white populations in European countries and North America, impeding the customization of Alzheimer’s approaches for individuals of diverse ethnic backgrounds.^7^

## Hypothalamus–Pituitary–Adrenal Axis

The hypothalamus–pituitary–-adrenal(HPA) axis has been thoroughly studied following its activation by stress, including in the pathophysiology of AD, where increased levels of circulating cortisol have been observed, as demonstrated in a recent meta-analysis.^8^ Also the activity of the HPA axis alters the makeup of the gut microbiota and enhances intestinal permeability, allowing bacterial substances to enter the bloodstream and thereby initiating a state of chronic low-grade inflammation. In a study, consumption of the Bifidobacterium longum 1714 strain by healthy human volunteers led to a reduction in cortisol levels. Concurrently, this psychobiotic with translational potential enhanced visuospatial memory performance dependent on the hippocampus. Prolonged exposure to glucocorticoids (GCs) or stress also triggers additional related pathogenic processes and dysfunctions, such as neuronal remodeling and deficits in synaptic function. The glial cells in the brain play crucial roles in regulating neuroinflammation and maintaining neuronal balance. A recent study highlighted their reciprocal signaling, demonstrating their ability to initiate a robust immune response. These glial cells express significant levels of mineralocorticoid receptor and glucocorticoid receptor, both of which are targets for the action of GCs. Consequently, astrocytes undergo morphological, functional, and transcriptomic changes following stress and activation of the HPA axis.^9^ Directing the cannabinoid system through modulation of endogenous mechanisms or external activation of cannabinoid receptors using natural or synthetic compounds found in cannabis has emerged as a promising approach for intervening in AD.

## Hypothalamus–Pituitary–Gonadal Axis

Hypothalamic–pituitary–gonadal (HPG) hormones play a role in regulating neuronal development and diverse brain functions. These hormones’ receptors are situated in memory and learning centers in the brain, such as the hippocampus (Table 1). For example, ovarian hormones control the turnover of synapses in the CA1 region of the hippocampus during the 4- to 5-day estrous cycle in female rats. Estradiol triggers the formation of new excitatory synapses through N-methyl-D-aspartate receptors, while the reduction of these synapses involves intracellular progestin receptors.^10^

Changes in HPG hormones associated with aging have been connected to the risk of AD. Moreover, research utilizing treatments targeting the disrupted signaling of the HPG axis has demonstrated significant enhancements in cognition and reductions in the progression of AD pathology.^11^ It was shown that there is a lower occurrence and a postponement in the initiation of AD in both women and men who undergo hormone replacement therapy after menopause/andropause.^12^ Whether these effects result directly from estrogens and androgens or the partial inhibition of gonadotropin secretion due to the restoration of negative feedback in the pituitary/hypothalamus remains unclear and requires further investigation.

## Gut–Brain Axis

**Figure 2.**
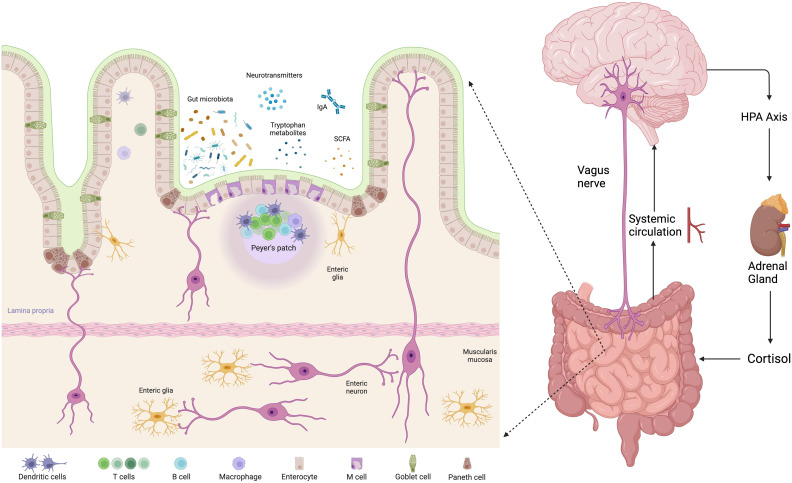
The gut and the CNS communicate bidirectionally through various direct and indirect pathways within the gut–brain axis. Communication pathways include the ANS (such as the ENS and the VN), the neuroendocrine system, the hypothalamic–pituitary–adrenal (HPA) axis, the immune system, and metabolic pathways. The gut microbiota generate a diverse range of neuroactive metabolites, especially neurotransmitters such as γ-aminobutyric acid (GABA), noradrenaline, dopamine, and serotonin (5-hydroxytryptamine). These compounds have the ability to pass through portal circulation in order to affect local ENS neuronal cells, afferent VN pathways that send signals directly to the brain, and the host immune system. Stress can trigger the HPA axis response in the nervous system, which involves hypothalamic neurons that secrete hormones into the brain or portal circulation, such as corticotropin-releasing hormone (CRH). This results in the release of adrenocorticotropic hormone (ACTH), which in turn starts the synthesis and release of cortisol. Intestinal barrier integrity is impacted by neuroimmune signaling responses, which are regulated by cortisol.

The gut–brain axis functions as a 2-way communication channel linking the gastrointestinal system and the brain. This interaction is facilitated through neuroanatomical routes such as the autonomic nervous system (ANS), the vagus nerve (VN), and the enteric nervous system (ENS).^13^ Additionally, it involves the HPA axis, the gut immune system, neurotransmitters, and neural regulators produced by gut microbiota, as well as the interplay between the intestinal mucosal barrier and the blood–brain barrier (BBB) (Figure 2).

From the perspective of the microbiome, which denotes the collective microorganisms inhabiting the gastrointestinal tract of humans, Firmicutes and Bacteroidetes stand out as the predominant phyla within the human intestinal microbiota.^14^ Dysbiosis, characterized by abnormal alterations in gut flora composition, is intricately linked to the pathophysiology of various diseases, including colorectal cancer and diabetes.^15^ A recent investigation utilizing a comprehensive multi-omics integrative approach uncovered significant variances in hub bacteria between AD and wild-type (WT) mice. Moreover, the study found strong associations between alterations in the gut microbiome and changes in fecal, serum, and cortical metabolomes observed in AD mice.^16^ Additionally, the introduction of gut microbiota from conventionally raised mutant amyloid precursor protein (APP)/presenilin 1 (APP/PS1) transgenic mice into germ-free mutant APP/PS1 transgenic mice resulted in a significant escalation of cerebral Aβ pathology in comparison to WT mice and germ-free transgenic mice.^17^

From a pathogenetic perspective, numerous studies have been conducted on the role of dysbiosis in the accumulation of tau and Aβ plaques. The mRNA expression of APP was discovered to be significantly elevated in the gastrointestinal tract compared to the brain. Conversely, the mRNA expression of tau was notably lower in the gastrointestinal tract compared to the brain.^18^ This phenomenon is largely supported by the antimicrobial properties exhibited by Aβ.^19^ While many studies have provided supportive data suggesting the enteric system as a precursor for B plaque accumulation, no study mechanistically elucidates its initiation.^20^ Another aspect to investigate is the mechanism by which the Aβ produced in the gastrointestinal tract is transported to the brain. Sun et al^21^ demonstrated the presence of Aβ deposits in the VN of mice injected with Aβ into the gastrointestinal tract. This supports the hypothesis proposing retrograde axonal transport via the VN, similar to what is suggested in prion and Parkinson’s diseases. However, while some researchers contend that transportation via the bloodstream could be equally significant,^22^ a study addressing this matter suggests that the relative contributions of each route vary depending on age.^18^ Hence, the causal connection between them remains unestablished. Further exploration is warranted into the potential of early manipulation of gut physiology and microbiota to reverse the pathology of AD.

In an alternative viewpoint, metabolites produced by bacteria—such as trimethylamine N-oxide, secondary bile acids (BAs), short-chain fatty acids (SCFAs), amyloid curli, and lipopolysaccharides—play a role in inducing various immune and metabolic changes that could potentially drive disease progression.^23^ Moreover, these metabolites may increase the permeability of both the intestinal and BBBs while also influencing inflammatory responses.

Interestingly, individuals with gastroesophageal reflux disease (GERD) are believed to have an increased likelihood of developing dementia compared to those without the condition. This is hypothesized to be linked to the reflux associated with GERD, which may alter the gastrointestinal microbiome. Additionally, the chronic damage to the tract and resulting inflammation could disrupt the regulation of cytokines within the body.^24^

In this context, modifications in the gut microbiota through diet, pharmacological interventions, probiotic or prebiotic supplementations, and fecal microbial transplantations hold promise as a therapeutic avenue for AD management. A research investigation has validated that maintaining a higher adherence to the Mediterranean diet may offer protection against brain aging and AD for 3.5 years, potentially diminishing the advancement of dementia.^25^ A significant breakthrough in pharmacological intervention comes in the form of GV-971, sourced from algae and comprising sodium oligomannate. This substance has shown considerable and consistent improvements in cognitive function during phase 3 clinical trials by notably reducing microglial activation and various brain proinflammatory cytokine levels. This effect is achieved through alterations in gut microbiota composition and a decrease in peripheral concentrations of phenylalanine and isoleucine produced by gut microbiota.^26^ The groundbreaking research conducted by Bonfili et al^27^ presented compelling evidence endorsing the beneficial impact of the probiotic formulation SLAB51 in mitigating cognitive decline, Aβ plaque formation, and brain damage. This effect is achieved by reinstating gut microbial balance in 3×Tg AD mice, thus offering a model for the therapeutic management of AD utilizing probiotics. A recent investigation indicates that prebiotics could potentially offer similar benefits in preventing and treating AD. Research on prebiotic therapy conducted in mice with AD revealed that yeast beta-glucan significantly increased the presence of both pro- and anti-inflammatory bacteria in the gut microbiota, boosted the production of SCFAs, and reduced neuroinflammation and brain insulin resistance.^28^ Lastly, regular transfer and transplantation of fecal microbiota from WT mice to ADLP^APT^ mice resulted in decreased generation of amyloid plaque and neurofibrillary tangle, reduced glial responses, and alleviated cognitive impairment. Fecal microbiota transplantation also reversed the abnormal expression of genes related to intestinal macrophage activity and reduced the elevation of circulating inflammatory monocytes in ADLP^APT^ recipient mice.^29^ In summary, while the approaches have shown potential for aiding in the treatment and prevention of AD, there remains a need for extensive clinical trial data on a larger scale and the development of standardized implementation strategies.

## Thyroid Dysfunction

**Figure 3.**
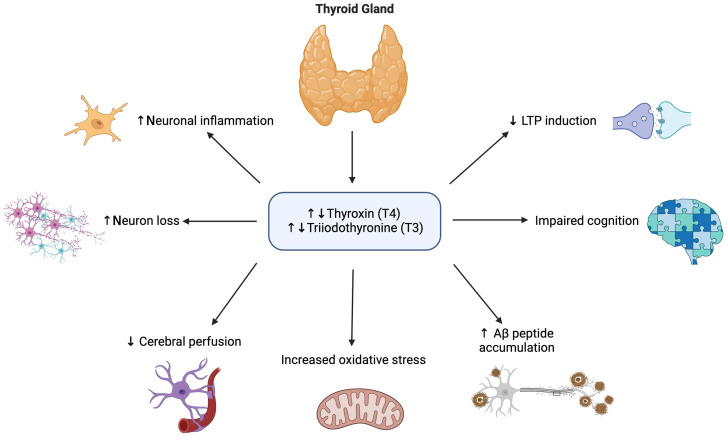
The effects of hypo/hyperthyroidism on AD.

Thyroid hormones (THs) are secreted by the thyroid gland and serve as vital regulatory molecules essential for vertebrate physiology and development. They play significant roles in the development of the fetal and postnatal nervous systems, as well as in maintaining the function of the adult brain. Cognitive impairment and dementia are linked to clinical thyroid disorders. Pre-clinical studies have indicated that aberrant signaling of THs leads to elevated levels of Aβ and increased tau phosphorylation.^30^ Several factors, including THs, have been demonstrated to influence the splicing and processing of AβPP. T3 not only controls the splicing of the *AβPP* gene but also regulates the processing and secretion of AβPP.^31^ A growing body of research has established the connection between learning and memory impairment, the onset of AD, and variations in TH levels and hypo- or hyperthyroidism.^30^ Similar effects on AD have been suggested for both hypo- and hyperthyroidism (Figure 3). Since hypothyroidism may be a risk factor for AD, AD itself may also cause dysregulation in the hypothalamus–pituitary–thyroid axis.

In a cross-sectional study by Choi et al,^32^ TH concentrations were associated with cerebral Aβ burden in euthyroid subjects. In another study utilizing prospectively gathered data from the Framingham Study, researchers aimed to clarify the relationship between thyroid function and dementia by investigating the likelihood of developing incident dementia and AD over a 12-year follow-up period in clinically euthyroid individuals. It was found that both low and high TSH levels were linked to a higher risk of incident AD among women, but not among men.^33^

## Heart–Brain Axis

Numerous cardiovascular risk factors have been consistently linked to an increased likelihood of subsequent cognitive decline in individuals without dementia.^34^ However, it is unclear whether atherogenesis develops concurrently with or separately from the accumulation of amyloid in the brain parenchyma in humans. Observational studies suggest that antihypertensive treatment may offer some advantages in lowering the concurrent occurrence of AD.^35^ The majority of antihypertensive medications commonly used in clinical settings affect the metabolism of APP/Aβ. Blocking the angiotensin-converting enzyme leads to elevated availability of Aβ1-40 or Aβ1-42 by reducing its breakdown, or by inhibiting the conversion of Aβ1-42 to Aβ1-40, respectively.^36^

Cerebral amyloid angiopathy (CAA) is a condition characterized by the buildup of Αβ1-40 deposits in the walls of the blood vessels in the brain. The tendency of Aβ1-40 to accumulate in blood vessels has led to the theory that this molecule may have proinflammatory effects not only in the brain but also in peripheral blood vessels.^37^ Although studies examining the relationship between plasma Aβ1-40 and cognitive function have produced inconsistent findings, Αβ peptides may be linked to vascular inflammation and extracerebral atherosclerotic conditions.

Interestingly, heart failure and AD have overlapping genetic and non-genetic factors, including hypercholesterolemia and metabolic syndrome. Furthermore, hypoxia, ischemia, and cellular stress can contribute to the elevation of BACE1 levels, and a deficit in energy leads to acidosis and oxidative stress in neurons, initiating a series of pathological events, including enzyme dysfunction and impaired protein synthesis resulting in the aggregation of both abnormal tau and Aβ proteins.^38^ Observational studies examining whether HF is a risk factor for AD yield conflicting results. Particularly, the inclusion of patients using antihypertensive medications in these studies, along with the known protective effects of antihypertensive drugs against AD, reduces the reliability of the findings.

## Liver–Brain Axis

In recent years, it has become increasingly obvious that chronic liver diseases extend beyond mere liver-focused conditions. Both animal models and clinical observations have revealed that chronic liver inflammation correlates with alterations in the CNS, leading to observable behavioral changes.^39^ The liver–brain axis mechanism primarily encompasses factors such as BBB permeability, the VN, epigenetic regulation, toxic metabolites, and Aβ metabolism. The BBB shows dysfunction in individuals with AD as well as in animal models. Further research is needed to explore the impact of BBB dysfunction on Aβ and to determine whether conditions affecting peripheral clearance also affect BBB function. Peripheral organs like the kidney and liver are crucial in removing circulating Aβ. Removing Aβ from circulation could potentially influence the progression of AD by shifting the equilibrium away from the buildup of Aβ in senile plaques toward soluble forms of Aβ. The inadequate removal of brain Aβ also plays a role in advancing sporadic AD. Hepatocytes can directly impact circulating Aβ by facilitating its removal through degradation or bile excretion. Additionally, the uptake of Aβ from circulation can occur via LRP-1, a receptor that is abundantly expressed in hepatocytes.^40^ The liver is regarded as a focal point for oxidative stress due to its role in regulating metabolism and inflammation, thereby supporting the immune system. Certain immune cells, including macrophages and lymphocytes, secrete proinflammatory molecules such as TNF-α and IL-1β. These molecules stimulate the secretion of secondary messengers like nitric oxide produced by cerebral endothelial cells, leading to changes within the brain.

In a similar way, TNF-α likely has a greater impact on the development of metabolic dysfunction-associated steatohepatitis (MASH) and fibrosis associated with metabolic dysfunction-associated fatty liver disease (MAFLD). Beyond being just a liver disease, various neurological symptoms of MAFLD and MASH have been reported, including reduced brain volume, subclinical or clinical cerebrovascular disease, and cognitive decline.^41^ Using both WT and AD transgenic mouse models, Kim and colleagues demonstrated that a slight increase in dietary lipids led to the development of MAFLD and acute hepatic inflammation, subsequently progressing to a chronic low-grade inflammatory condition. Additionally, the researchers observed an elevation in neuroinflammation, microgliosis, and astrogliosis, along with advanced indicators of AD such as accelerated generation of Aβ plaques, CAA, and increased tauopathy. In a recent study, it was shown that Aβ accumulation increased with the severity of MAFLD in females, while no correlation was observed between MAFLD and Aβ accumulation in males, indicating that treatments need to be developed specific to gender.^42^

There are some therapies targeting the liver–brain axis in animal models. Hepatic soluble epoxide hydrolase is one of the targeted candidates. In mammals, soluble epoxide hydrolase (sEH), a protein with an α/β hydrolase fold, is found in the cytosol of various tissues, including the liver, kidney, lungs, heart, brain, spleen, adrenal glands, and intestine, and occasionally in the peroxisome.^43^ Also, its function is implicated in the detoxification of xenobiotic epoxides. As individuals age, hepatic sEH activity increases. Manipulation of hepatic sEH levels has been shown to have dual effects on brain Aβ accumulation, tauopathy, and cognitive impairment in AD mouse models. Additionally, altering hepatic sEH levels has been found to regulate the plasma concentration of 14,15-epoxyeicosatrienoic acid (14,15-EET) that can efficiently cross the BBB and influence Aβ metabolism in the brain through various pathways. Maintaining a balance between the levels of 14,15-EET and Aβ in the brain is crucial for preventing Aβ deposition.

## Obesity

Obesity has been associated with cognitive impairments, reduced long-term potentiation and synaptic plasticity, as well as diminished brain volume, raising the risk of AD and other forms of dementia. Obesity induces a persistent low-grade inflammatory condition, which is a common feature of several chronic conditions including metabolic syndrome, nonalcoholic fatty liver disease, type 2 diabetes mellitus, and cardiovascular disease. Additionally, it contributes to neuroinflammation, which is a key feature of neurodegenerative disorders like AD.^44^ However a meta-analysis indicated a positive correlation between midlife obesity and future dementia, while the opposite was observed in late life.^45^ Furthermore, an analysis of data from 1.3 million individuals suggested that while higher body mass index (BMI) had detrimental effects over the long term, it seemed to offer protection in the short term before dementia diagnosis. These findings support the notion of an “obesity paradox” in dementia, originally proposed in heart failure, where obese patients had lower mortality rates despite increased risks of vascular diseases.

In a recent study, Suzzi et al^46^ suggested that the connection between obesity and the susceptibility to AD may be attributed to systemic immune exhaustion. In an obese AD mouse model (5×FAD mice), there were minimal diet-related transcriptional alterations observed in hippocampal cells, while the immune composition in the spleen exhibited deregulation of CD4+ T-cells resembling aging. Through plasma metabolite profiling, they discovered that elevated levels of free N-acetylneuraminic acid, the primary sialic acid, correlated with recognition-memory impairment and an increased presence of immune-suppressive cells in the spleen of mice.

Leptin, encoded by the Ob gene, is one of central molecules in energy homeostasis. As the number of studies demonstrating the relationship between obesity and AD increases, the connection between leptin, which plays a role in obesity, and Alzheimer’s has begun to attract attention.^47^ Significantly, studies have revealed a correlation between serum leptin levels and the onset of AD, indicating a possible pathogenic role for leptin.

APOE4 is one of the main genetic predisposing factors for the onset of AD in later stages of life. Outside of the brain, APOE4 is linked to a higher likelihood of developing metabolic syndrome and cardiovascular disease.^48^ Individuals carrying the APOE4 allele exhibit notably elevated levels of fasting glucose and insulin, as well as a heightened risk of metabolic syndrome with an earlier onset age. These observations were made independent of any significant increase in BMI.^49^ However the relationship between obesity and APOE genotypes is not as well-defined. Several investigations have explored the metabolic and cognitive impacts of APOE genotypes and obesity separately. There is limited research that simultaneously examines the effects of a high-fat diet on both metabolism and cognition under identical conditions concerning age and gender. Further extensive research is necessary to investigate metabolic changes and cognitive functions in obese individuals with various APOE genotypes.

## Sensorial Loss

We engage with the physical world through our senses, which support our behavioral performance and various life activities. Sensorial loss plays a complex role in the pathogenesis of AD, acting both as a potential cause and a consequence of the disease’s progression. On one hand, sensorimotor deficits can contribute to the development and exacerbation of AD by impairing cognitive functions through reduced sensory input and motor coordination.^50^ Disruptions in sensory processing may affect spatial navigation and memory consolidation, which are critical for cognitive function and often deteriorate in AD. A study by Shen et al^51^ induced deafness in mice with ototoxic drugs and showed that this hearing loss resulted in hippocampal neuron degeneration, which was linked to increased inflammation and higher levels of p-tau. In another study, it was observed that hearing loss was independently associated with the onset of dementia involving 639 participants. Additionally, the attributable risk of dementia related to hearing loss was found to be 36.4%.^52^

Patients with AD have been reported to experience a range of visual issues, including reduced visual acuity, impairments in color vision and visual fields, altered pupillary responses to mydriatics, defects in fixation, as well as in smooth and saccadic eye movements, and changes in contrast sensitivity.^53^ Besides, several recent studies have found Aβ plaques, a key characteristic of AD, in the retinas of both AD mouse models and humans.^54^ Recently, Lee et al^55^ discovered that individuals with visual impairment had a substantially increased risk of developing all types of dementia over a 12-year follow-up period. Remarkably, the risk was nearly twice as high in young males compared to older females.

Impairment in olfactory identification may occur before noticeable cognitive impairments become evident. It has been demonstrated that the link between olfactory dysfunction and clinical AD is primarily attributed to the buildup of AD’s pathology, particularly neurofibrillary tangles, in central olfactory areas, notably the entorhinal cortex and hippocampus.^56^ From a different perspective, Laukka et al^57^ showed that olfactory dysfunction is associated with an increased risk of dementia in both younger and older adults, regardless of sex. Severe olfactory dysfunction, particularly when combined with the APOE4 allele, is associated with a significantly increased risk of developing dementia.

## Future Directions

Detecting AD early is crucial, and finding dependable biomarkers using minimally invasive methods remains a significant obstacle in the field. Further exploration of peripheral biomarkers could provide valuable tools for early detection, diagnosis, and monitoring of AD. Research efforts should focus on identifying reliable markers in blood, cerebrospinal fluid, and other bodily fluids that reflect AD-related pathologies in the brain. Additionally, developing interventions that target both central and peripheral aspects of AD pathology could offer synergistic benefits. Multimodal therapies that simultaneously address brain Aβ deposition, neuroinflammation, and peripheral dysfunction may hold promise for slowing disease progression and improving clinical outcomes. Furthermore, considering the varying outcomes of similar studies, consolidating study results in a single center for analysis across a larger population will significantly enhance the reliability of the data.

It is crucial to recognize that understanding AD requires more than just expertise from 1 discipline alone. By collaborating across various fields and gathering the most reliable and significant information, researchers can embark on novel initiatives that were previously unthinkable. This collaborative effort will be essential for unraveling the intricate puzzle of AD and will provide a broader perspective on the issue.

## Conclusion

The discussion of AD encompasses a broad range of challenges and potential avenues for progress. Firstly, the failure of drug treatments targeting Aβ plaques and tau protein abnormalities in late-stage clinical trials underscores the need for a deeper understanding of AD pathophysiology. This prompts critical questions regarding our current understanding of the disease and the potential need to explore alternative therapeutic approaches. Additionally, the recognition of AD as a systemic condition involving communication between the brain and peripheral organs opens new avenues for research. While studies suggest that peripheral processes may influence or be influenced by AD pathology, the exact mechanisms remain elusive. However, exploring the role of peripheral organs in AD pathogenesis holds promise for uncovering novel therapeutic targets and interventions. Addressing these challenges requires interdisciplinary collaboration and innovative research strategies to advance our understanding of AD and develop effective treatments.

## Figures and Tables

**Table 1.** Hypothalamic–Pituitary–Gonadal (HPG) Hormones Have Different Functions in Different Brain Regions

Receptor	Localization	Type	Function	References
GnRH receptors	Hippocampus, cerebral cortex, basal ganglia, spinal cord and cerebellum.	Rhodopsin-like G-protein coupled 7-transmembrane domain receptors.	Modulation of motor function, modification of memory and reproduction.	10
Gonadotropin receptors	Hippocampus, brainstem, cortex, choroid plexus, and pituitary gland.	G-protein coupled 7-transmembrane domain receptors.	Neuronal development and maintenance, neurosteroidogenesis, sensory information, processing.	10
Sex steroid receptors	Hippocampus, amygdala, cerebellum.	Nuclear and transmembrane receptors.	Neuroprotection, emotion processing, reward processing, cognition.	10
Activin receptors	Hippocampus, amygdala, cerebral cortex, and thalamus.	Heteromeric combinations of type II (ActRIIA, ActRIIB) and type I receptors (primarily ActRIB, along with some ActRIA and ActRIC).	Regulation of spine formation, adult neurogenesis, maintenance of late-phase LTP (long term potentiation) memory consolidation.	10

LTP, long-term potentiation.

## Data Availability

The data that support the findings of this study are available on request from the corresponding author.
